# Evolutionary Mechanism Based Conserved Gene Expression Biclustering Module Analysis for Breast Cancer Genomics

**DOI:** 10.3390/biomedicines12092086

**Published:** 2024-09-12

**Authors:** Wei Yuan, Yaming Li, Zhengpan Han, Yu Chen, Jinnan Xie, Jianguo Chen, Zhisheng Bi, Jianing Xi

**Affiliations:** School of Biomedical Engineering, Guangzhou Medical University, Guangzhou 511436, China; yuanwei@gzhmu.edu.cn (W.Y.); felicefy1229@gmail.com (Y.L.); m18933690693@163.com (Z.H.); yuchenmad@outlook.com (Y.C.); ys1442327007@163.com (J.X.); cjg3135056801@outlook.com (J.C.)

**Keywords:** Conserved Gene Expression Module, biclustering, evolutionary mechanism, breast cancer, Mean Squared Residue Score

## Abstract

The identification of significant gene biclusters with particular expression patterns and the elucidation of functionally related genes within gene expression data has become a critical concern due to the vast amount of gene expression data generated by RNA sequencing technology. In this paper, a Conserved Gene Expression Module based on Genetic Algorithm (CGEMGA) is proposed. Breast cancer data from the TCGA database is used as the subject of this study. The *p*-values from Fisher’s exact test are used as evaluation metrics to demonstrate the significance of different algorithms, including the Cheng and Church algorithm, CGEM algorithm, etc. In addition, the F-test is used to investigate the difference between our method and the CGEM algorithm. The computational cost of the different algorithms is further investigated by calculating the running time of each algorithm. Finally, the established driver genes and cancer-related pathways are used to validate the process. The results of 10 independent runs demonstrate that CGEMGA has a superior average *p*-value of 1.54 × 10^−4^ ± 3.06 × 10^−5^ compared to all other algorithms. Furthermore, our approach exhibits consistent performance across all methods. The F-test yields a *p*-value of 0.039, indicating a significant difference between our approach and the CGEM. Computational cost statistics also demonstrate that our approach has a significantly shorter average runtime of 5.22 × 10^0^ ± 1.65 × 10^−1^ s compared to the other algorithms. Enrichment analysis indicates that the genes in our approach are significantly enriched for driver genes. Our algorithm is fast and robust, efficiently extracting co-expressed genes and associated co-expression condition biclusters from RNA-seq data.

## 1. Introduction

The quest to understand cancer at the molecular level reveals a complex landscape where cancer cells demonstrate unique gene expression patterns diverging significantly from their healthy counterparts. The advent of high-throughput sequencing technologies, such as RNA sequencing (RNA-seq), has opened new vistas in cancer research by furnishing an unparalleled richness of transcriptomic data [1,2]. These technologies have catapulted us into an era where the voluminous and sophisticated nature of genomic data presents a formidable challenge, necessitating the use of computational techniques to sift through this extensive dataset to unearth genes that exhibit coordinated expression under specific conditions [3,4]. Concurrently, it has become increasingly clear that cancer is not a consequence of anomalies in single genes but emerges from complex interactions among multiple co-expressed RNAs.

Amidst this complexity, it is crucial to identify the specific conditions under which cancer genes are co-expressed. The specific conditions of co-expression, which leads to the research question of biclustering. Biclustering allows us to identify clusters of genes that exhibit coordinated expression under certain conditions, offering insights that conventional clustering methods, which assume co-expression across all conditions, might miss [5,6,7,8,9,10,11,12]. The quest for deciphering these patterns has spurred the development of various heuristic algorithms since the pioneering CC algorithm by Cheng and Church in 2000 [13]. These include the Modified Cheng and Church’s algorithm (MCC) [14], the Large Average Submatrix algorithm (LAS) [15], the Relative Density based Biclustering Method (RelDenClu) [16], and the Connectedness-based subspace clustering (CBSC) [17], all designed to tackle the biclustering challenge as an optimization problem, striving for efficient extraction of meaningful genomic insights from RNA-seq data [18,19]. In the comparative analysis of biclustering algorithms, each method presents distinct characteristics that allow it to be applied to specific research needs in a tailored manner. The spectral CC method is particularly effective in identifying co-expressed gene and condition submatrices, which is a valuable capability in the analysis of gene expression data. However, its high time complexity and sensitivity to noise can act as limitations, particularly when dealing with large datasets. MCC offers an ensemble learning approach that enhances stability and accuracy across various data types, with robustness against noise, although this is at the cost of higher computational requirements. LAS proposes a statistical method that is effective in detecting numerically significant submatrices within high-dimensional data, demonstrating strong resistance to noise and scalability to large datasets, despite its computational intensity. Conserved Gene Expression Motif (CGEM) employs a graph-based approach to identify conserved gene expression motifs, offering insights into gene regulatory networks [20]. However, its utility is more suited to medium to small-scale datasets, and its effectiveness depends on the stability of the graph structure. RelDenClu and CBSC are density-based subspace clustering algorithms that aim to partition data points into multiple subspaces, wherein data points within the same subspace exhibit higher connectivity compared to those in different subspaces. RelDenClu identifies subsets of observations exhibiting dependence between features by comparing joint and marginal densities, and subsequently groups data points based on these features. In contrast, CBSC leverages connectivity scores to identify subspaces. Both algorithms exhibit robustness, although CBSC is more computationally intensive. RelDenClu is capable of uncovering feature relationships based on nonlinear dependencies, whereas CBSC is more suited for linear relationships. In conclusion, the selection of an appropriate biclustering algorithm should be based on the characteristics of the data set, the specific research objectives, and the available computational resources. Each algorithm possesses distinctive advantages in identifying the intrinsic patterns within biological data. Table 1 provides a comparative analysis of the aforementioned six algorithms.

Although these algorithms represent a significant advance in the field, they are not without limitations. In particular, their reliance on exhaustive traversals to identify genetic traits associated with cancer renders them somewhat inefficient. This process is both time-consuming and a significant consumer of computing resources [21,22,23]. Furthermore, the complex interrelationships between diverse gene combinations and cancer progression present a significant challenge to optimization, particularly when using traditional methods [24]. However, recent advances have demonstrated the potential of evolutionary algorithms (EAs) in overcoming these challenges. They exhibit robust global optimization capabilities, parallel processing properties, adaptability and resilience, maintenance of population diversity, adaptive tuning of parameters, scalability of algorithms, and a search process that does not depend on predefined thresholds. These characteristics assist the genetic algorithm in circumventing local optimal solutions, thereby enhancing the algorithm’s efficacy in processing extensive gene expression data and offering a new paradigm for efficient bicluster identification.

Leveraging this insight, we introduce the Conserved Gene Expression Module algorithm enhanced by a Genetic Algorithm (CGEMGA). This novel approach not only capitalizes on the strengths of the CGEM algorithm but also harnesses the power of EAs to navigate the optimization landscape more effectively. By incorporating the Mean Squared Residual Score (MSR) criterion, CGEMGA stands out in its ability to efficiently identify the most optimal gene combinations pertinent to breast cancer, as validated by rigorous testing against breast cancer datasets, and Fisher’s exact test comparisons with other algorithms. Remarkably, this approach not only enhances reliability but also drastically reduces computational overhead, setting a new benchmark in biclustering algorithm efficiency.

## 2. Materials and Methods

### 2.1. Data Acquisition of Breast Cancer Samples

In our quest to unravel the complex genetic architecture of breast cancer, we have procured gene expression profiles from The Cancer Genome Atlas (TCGA). This global repository offers an extensive compendium of human cancer genomes, serving as a beacon for researchers worldwide [25,26,27,28]. Our analysis is anchored in the rich dataset of 421 breast cancer samples, encompassing a diverse array of 12,129 genes, carefully selected for their relevance to our study.

To further reinforce the empirical foundation of our study, we refer to the Catalogue of Somatic Mutations in Cancer (COSMIC) as a reference point. The Cancer Gene Census (CGC) on the Sanger Institute’s website provides access to the comprehensive and meticulously curated catalogue of cancer-driving genes, COSMIC. This database, which is instrumental in both cancer genetics research and drug development initiatives, has recently been expanded to encompass 739 genes that are crucial to the progression of cancer [29,30]. The judicious choice of these datasets shows how seriously we’re taking the molecular intricacies of breast cancer.

### 2.2. Overview of Biclustering

The concept of biclustering, a term coined by Cheng and Church in the groundbreaking introduction of their CC algorithm in 2000, has evolved significantly over the years. This pioneering contribution heralded a new era in the exploration of gene expression dynamics across diverse conditions through the application of biclustering techniques. These sophisticated algorithms, by framing the biclustering challenge as an optimization problem (COP), set out to uncover patterns within the genetic matrix that elude traditional analysis methods. At the heart of this endeavor is the quest for submatrices within the gene expression matrix that exhibit unique patterns of interest, encapsulated in the specially defined score functions and heuristic solutions of the COP [31,32].

To illustrate, let’s consider the data matrix X={E,F}, where $E=\{e_{1},\ldots e_{N}\}$ symbolizes the set of N genes and $F=\{f_{1},\ldots ,f_{M}\}$ represents the set of M conditions. Within this framework, a bicluster B emerges as a subset, defined by $B=\{\left(E_{B},F_{B}\right);E_{B}\subseteq E,{\;F}_{B}\subseteq F\}$. This subset transcends to the status of a bicluster only if it adheres to specific patterns, typically quantified by the Mean Squared Residual (MSR) score.

The MSR is defined as:

(1) $$H\left(I,J\right)=\frac{1}{\left|I\right|\left|J\right|}\sum_{i\in I,j\in J}{\left(a_{ij}-a_{iJ}-a_{Ij}+a_{IJ}\right)}^{2},$$

where $a_{ij}$ denotes the matrix element, with the row and column means and the overall mean of the submatrix B calculated as follows:

(2) $$a_{iJ}=\frac{1}{\left|J\right|}\sum_{j\in J}a_{ij},\;\;a_{Ij}=\frac{1}{\left|I\right|}\sum_{i\in I}a_{ij},\mathrm{and}\;a_{IJ}=\frac{1}{\left[I\right][J]}\sum_{i\in I,j\in J}a_{ij}=\frac{1}{\left[I\right]}\sum_{i\in I}a_{iJ}=\frac{1}{\left[J\right]}\sum_{j\in J}a_{Ij}.$$

This mathematical rigor provides a robust framework to unravel the complex patterns of gene expression specific to breast cancer, laying the groundwork for a deeper understanding of the disease’s genetic architecture.

### 2.3. Conserved Biclustering Algorithm

At the heart of our exploration into the genetic underpinnings of breast cancer lies the Conserved Gene Expression Motif (CGEM), an innovative biclustering algorithm. This method is grounded in a simple yet profound principle: if a gene maintains consistent expression across a subset of samples, its expression level is considered conserved within that specific subset [33]. This approach diverges markedly from traditional algorithms such as CC, which rely heavily on scoring schemes to identify significant patterns. Instead, CGEM seeks out conserved gene modules across the entirety of the dataset, employing constraints to uncover these vital connections [34].

The CGEM algorithm distinguishes itself by its ability to unearth the largest xmotif, representing a conserved gene expression pattern of paramount significance. This endeavor involves a meticulous, iterative process composed of three fundamental steps. Initially, the algorithm identifies the most extensive xmotif within the dataset, guided by a designated seed *s*. This identification process prioritizes the discovery of motifs with a substantial number of conserved genes. Following this, it strategically removes the samples aligning with this motif from consideration, thereby refining the dataset. The subsequent step involves a renewed search within this pared-down dataset, aiming to locate the next largest motif. This iterative loop continues until the algorithm selects the submatrix with the largest row-column size among all potential biclusters.

This iterative, constraint-based methodology sets CGEM apart, offering a novel lens through which to view the complex landscape of gene expression in breast cancer. By focusing on the conservation of gene expression across samples, CGEM provides invaluable insights into the genetic consistency that may underpin this disease, offering promising avenues for further research and potential therapeutic targets.

### 2.4. Evolutionary Mechanism-Based Conserved Gene Expression Biclustering Module

While the CGEM algorithm marks a significant leap forward in identifying conserved gene modules, it harbors certain limitations that hinder its potential for global optimization. These challenges include a predefined iteration count lacking mathematical justification, an absence of a fitness function for optimal module evaluation, and the arbitrary selection of the initial seed s, which compromises the pursuit of the global optimum [35]. Additionally, despite the high accuracy of many biclustering algorithms, their extensive exploratory capabilities contribute to increased computational demands [21,23].

To surmount these obstacles, we introduce the CGEMGA (Conserved Gene Expression Module Genetic Algorithm), an innovative approach that incorporates an evolutionary algorithm to navigate toward globally optimal biclustering. The genetic algorithm (GA), inspired by Darwin’s principle of natural selection, serves as the cornerstone of our method, utilizing population-based strategies and the survival of the fittest principle to refine the search for optimal gene modules [36]. Unlike the CGEM algorithm, which relies on randomly selected seed s, our approach optimizes the initial seed selection through GA mechanisms, including population genetics, crossover, and mutation. This strategy enables the identification of an optimal seed s by evaluating the MSR of submatrices generated from each seed and selecting the one with the minimal MSR value, thereby ensuring the selection of the most stable module.

Outlined in Table 2, our algorithm’s process begins with the input of genes, samples, and their expression values, alongside intervals representing gene expression states. Assuming distinct intervals for each gene’s states, the algorithm uniformly selects $n_{s}$ sample groups from the entire sample set. In contrast to the CGEM algorithm’s random seed generation, our method employs GA to ascertain optimal seeds through a meticulously defined procedure:

1. Initiate with a population of n chromosomes $C_{i}$ (i=1,2,…,n) as potential seeds s.
2. For each chromosome $C_{i}$, identify a subset of samples $D_{i}$ of size $s_{d}$.
3. Include gene-sample pairs (g,s) in set $G_{ij}$ if gene g exists in state s across all samples in $D_{i}$, and also incorporate samples matching c across all gene states in $G_{ij}$.
4. Compute the MSR fitness value for each chromosome.
5. Apply GA’s selection, mutation, and crossover operations to optimize based on the MSR fitness value, thereby deriving the optimal solution.
6. Exclude any $C_{ij}$ representing less than a fraction α of the samples.
7. Select the module with the lowest MSR from all $C_{i}$ as the final choice.

**Figure 1:**
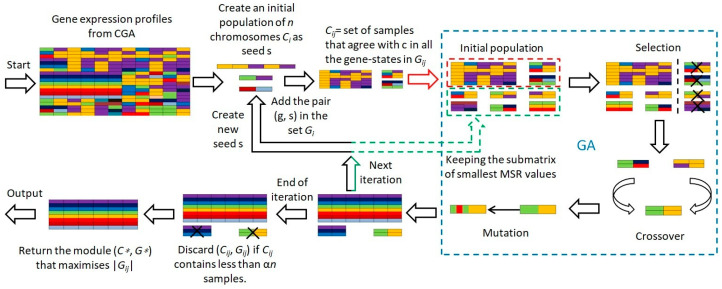
Flowchart of the CGEMGA algorithm.

Employing GA to refine the initial seed s and evaluating modules via MSR during each iteration not only circumvents the fixed iteration constraint of the CGEM algorithm but also significantly reduces computational time, showcasing the efficacy of our evolutionary approach. Figure 1 illustrates the flowchart of our algorithm.

### 2.5. Evaluation Metrics

In the complex process of validating our evolutionary mechanism-based conserved gene expression biclustering module, precision in detecting breast cancer genes stands paramount. To achieve this, we juxtapose our method against both the CGEM algorithm and other prevailing algorithms through a series of ten meticulously conducted independent runs. Post each execution, the generated results undergo a rigorous comparison with the breast cancer gene data cataloged in COSMIC. This comparative analysis serves not only as a validation of our method’s effectiveness but also as a critical link that bridges theoretical advancement with empirical confirmation.

The cornerstone of our evaluation lies in the application of Fisher’s exact test, a statistical method renowned for its precision in assessing the association between the outcomes of our algorithm and the presence of breast cancer genes. The Fisher exact test embarks on this task by meticulously calculating *p* values, predicated on the analysis of all conceivable configurations of 2 × 2 contingency tables that manifest marginal totals equivalent to or surpassing those observed. The test’s foundation is built upon a sample drawn from a population of size N, with m objects exhibiting trait A (a within the sample and c outside the sample) and n objects not exhibiting trait A (b within the sample and d outside the sample). Here, the aggregates of a and b form r, while those of c and d constitute s. The calculation of the *p*-value unfurls through the following formula:

(3) $$p=\frac{m!n!r!s!}{a!b!c!d!N!}.$$

Delving deeper into the specifics, our application of Fisher’s exact test involves the enumeration of genes identified by the biclustering algorithm and present in the CGC dataset as a, juxtaposed with genes identified by the algorithm but absent in the CGC dataset as b. Concurrently, we account for genes present in the CGC dataset yet overlooked by the biclustering algorithm as c, and genes neither detected by the algorithm nor listed in the CGC dataset as d. A threshold of p<0.05 delineates the boundary for statistical significance, guiding us in discerning meaningful associations.

The essence of our study is encapsulated within the realm of unsupervised learning models, which inherently operate without the crutch of predefined labels, thus obviating the hurdles of data partitioning. This attribute, while liberating, mandates the necessity for external validation through third-party corroborations. In our quest for validation, we anchor our trust in established cancer driver genes and related pathways, leveraging these benchmarks not just as a means of validation but as a beacon guiding our exploratory voyage through the genomic landscape of breast cancer.

## 3. Results

### 3.1. Experiment Setup

To examine the effectiveness of biclustering data searching of our CGEMGA algorithm, we conducted several experiments to analyze our approach from multiple perspectives: (1) investigating the superiority between our approach and CGEM algorithms by comparing the *p-*values of Fisher’s test with the MSR values [37,38,39]; (2) comparing our approach with widely-used existing biclustering approaches; (3) testing the computational cost of the entire algorithm’s runtime; (4) studying the quantitative differences between our approach and the CGEM algorithm by the F-test [40]; (5) unscrambling gene functions by enrichment analysis.

The experimental apparatus was manufactured by Lenovo in Shenzhen, China, and was powered by an Intel Xeon(R) CPU E3-1225 v6 @ 3.30 GHz × 2 processors, backed by 32 GB of RAM. The datasets comprised 421 breast cancer samples, intricately mapped out across 12,129 genes. Leveraging unsupervised methods, we plunged directly into the data, eschewing any division between training, testing, or validation datasets, thus ensuring an unadulterated analysis.

### 3.2. Ablation Study

#### 3.2.1. Evolutionary Effect

To showcase our method’s superiority, we pitted it against the CGEM algorithm in ten independent trials, comparing their statistical significance and MSR values. Table 3 reveals a striking contrast: while the CGEM algorithm’s *p-*values fluctuate significantly, ours remain remarkably stable, underscoring the robustness of our approach. Specifically, CGEM’s *p-*values span from 9.62 × 10^−6^ to 9.71 × 10^−2^ averaging at 1.18 × 10^−2^ ± 3.01 × 10^−2^. The MSR variations mirror this volatility. In stark contrast, our method consistently delivers *p* values with minimal variance, showcasing not only our algorithm’s precision but also its reliability, a testament to the evolutionary mechanics at its core.

#### 3.2.2. Stability Analysis

Venturing further, we employed the F-test to quantitatively assess the differences between our algorithm and CGEM, seeking to cement the stability and reliability of our findings. The F-test, executed with SPSS, yields an F-value of 4.940, decisively surpassing the threshold of 3.18. This, coupled with a *p*-value of 0.039, significantly below the conventional benchmark of 0.05, unequivocally confirms the superior performance and stability of our approach over CGEM.

#### 3.2.3. Computational Cost

**Figure 2:**
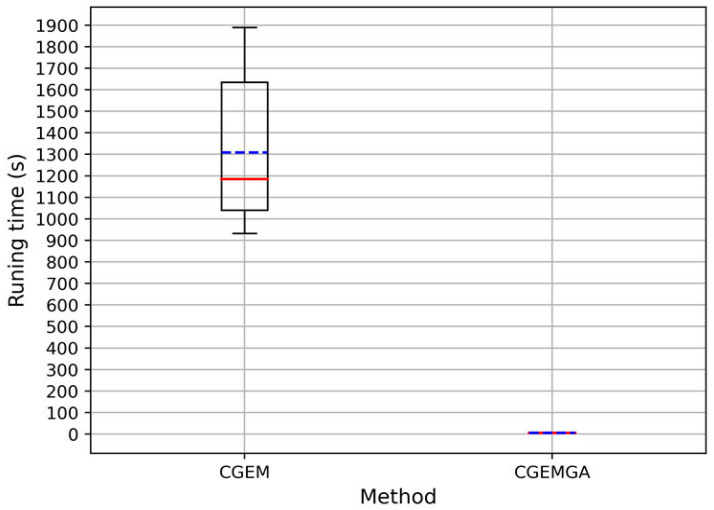
Runtime of CGEM and CGEMGA in seconds (the solid red line in the figure represents the median value, while the blue dashed line represents the mean value).

In the realm of computational efficiency, our method shines bright, dramatically outpacing CGEM and other evaluated algorithms. An analysis of runtimes across ten trials showcased CGEM’s considerably longer durations, with an average runtime dwarfing ours. Our method clocked in at an astonishingly low average of 5.22 ± 1.65 × 10^−1^ s (Figure 2 and Figure S1 and Table 4), a mere fraction of CGEM’s, thus not only illustrating our approach’s swiftness but its unparalleled efficiency and stability in the face of complex genomic data.

### 3.3. Comparison Study

In order to obtain a comprehensive assessment of the identification performance, an analysis of the most widely used biclustering approaches is conducted, with a comparison to our approach made using Fisher’s exact test *p*-values. Through meticulous analysis, juxtaposed against the rigorous benchmarks set by Fisher’s exact test *p-*values, we sought to illuminate the distinctive prowess of our approach. The tableau of results, as detailed in Table 5, unveils a panorama of statistical variance across the algorithms. Notably, the MCC algorithm’s *p-*values oscillated broadly, marking a contrast against the more consistent yet equally varied performance of the CC and LAS algorithms. The RelDenClu and CBSC algorithms yield *p-*values that are relatively consistent and demonstrate superior performance compared to the MCC, CC, LAS, and CGEM algorithms. Amidst these statistical results, our CGEMGA algorithm emerged as a beacon of stability and precision, boasting the lowest average *p-*value with unparalleled consistency.

**Figure 3:**
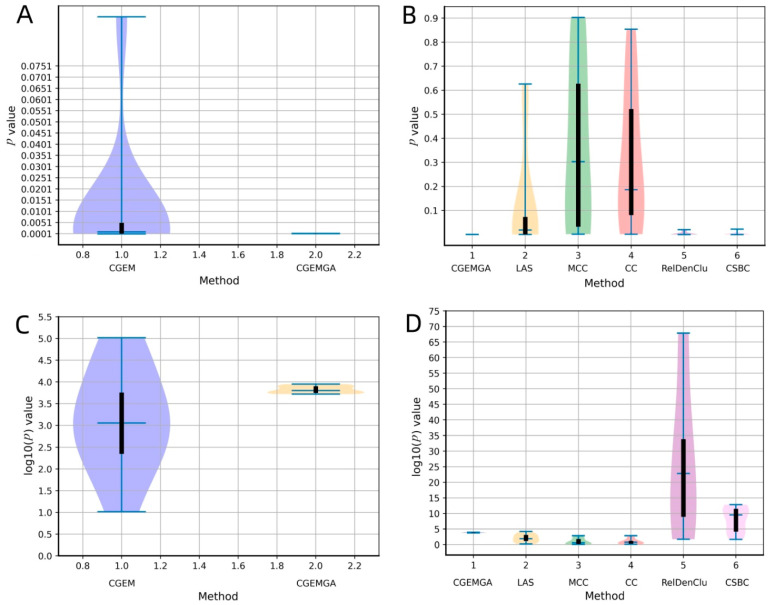
The comparison of *p-*values between different methods. Where (**A**) stands for *p* values comparison between our approach and CGEM, and (**B**) stands for *p* values comparison between our approach and RelDenClu, CBSC, LAS, MCC, and CC. (**C**,**D**) are negative log10 operations on the *p* values in the (**A**,**B**).

**Figure 4:**
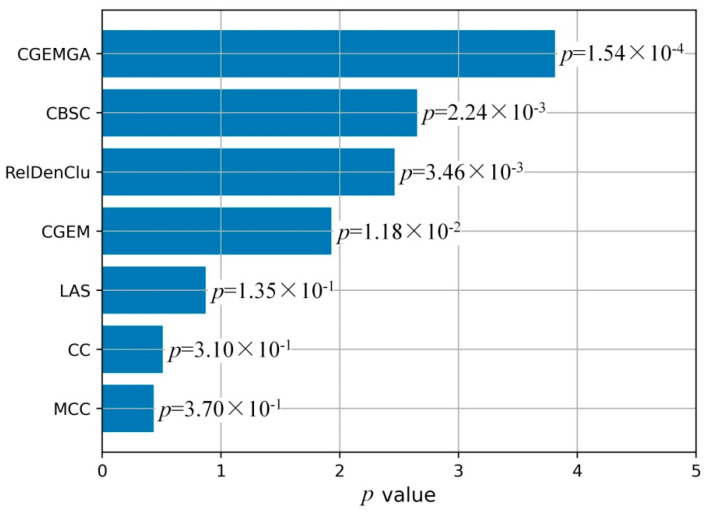
Comparison of mean *p-*value of five methods.

To visually articulate these findings, Figure 3 unfolds in a duo of plots, each casting our algorithm in a comparative light against both the CGEM algorithm and the ensemble of established biclustering methods. Further enriched by plots C and D, which transform these *p-*values into a more visually impactful negative log10 scale, our narrative of superiority is vividly underscored. Figure 4, echoing this theme, presents a compelling graphical representation of our method’s statistical dominance, showcasing the smallest *p-*values amidst a backdrop of high stability and consistency, significantly outshining the comparative algorithms.

**Figure 5:**
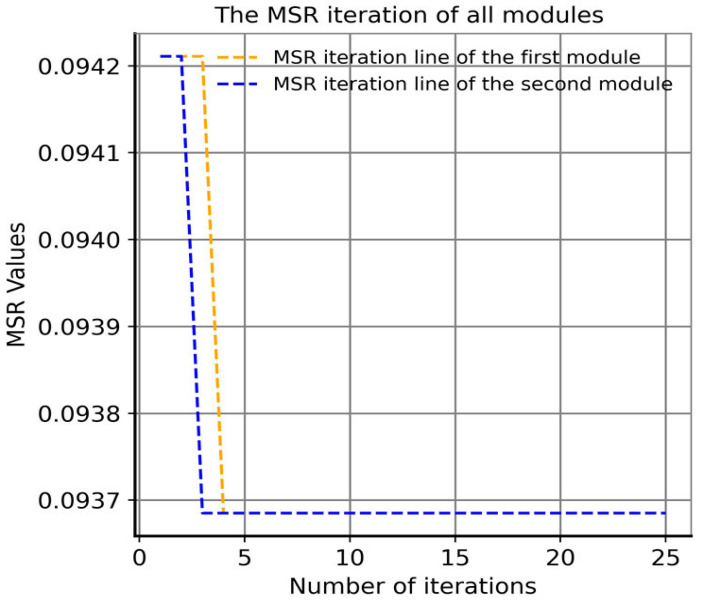
Iterative convergence curve of the genetic algorithm with MSR as a fitness function in our approach.

Moreover, in order to examine the convergence of the GA proposed by our approach, we undertake an analysis of the number of iterations required for convergence. The algorithm is designed to generate, on average, two modules per iteration. As illustrated in Figure 5, both modules converge to the global optimal solution value of 9.37 × 10^−2^ in less than five iterations, indicating a rapid convergence rate.

### 3.4. Functional Enrichment Analysis

While our algorithm’s prowess in navigating the complex genomic landscape of breast cancer is undeniably impressive, the true essence of this journey lies in unraveling the biological narratives of the genes thus identified. Functional enrichment analysis emerges as a vital tool in this quest, bridging the gap between gene clusters and their biological functions. By leveraging the DAVID platform, a beacon in the bioinformatics realm, we not only validate the biological significance of the identified genes but also illuminate the pathways they orchestrate. Our analysis, capturing forty-five genes within the TCGA breast cancer dataset (Table 6), reveals a rich tapestry of functional associations, underscoring the enriched biological relevance of these gene modules (Table 7).

A closer examination reveals further fascinating insights. For instance, the role of the *CDH1* gene in lobular breast cancer is well documented [41,42], while the *FEN1* gene is associated with poor prognoses [43]. Additionally, the complex interactions between *POLQ* and *TP53* have been extensively studied [44,45]. These accounts, substantiated by meticulous research, not only corroborate the accuracy of our approach but also illuminate the complex interplay between genetics and cancer pathology.

## 4. Discussion and Conclusion

In the complex field of breast cancer genomics, we propose a state-of-the-art biclustering algorithm that draws inspiration from the adaptive power of genetic algorithms (GA). At the core of our exploration, utilizing the robust analytical frameworks of Fisher’s exact test and the F-test, we meticulously scrutinized the performance of various algorithms against a backdrop of TCGA breast cancer data. This rigorous examination not only confirmed the superior significance of our method but also highlighted its computational agility and the depth of biological insights it unveils through functional enrichment analysis. With the F-test result (F = 4.940, *p* = 0.039) underscoring significant distinctions from the CGEM algorithm and a swift convergence within a mere five iterations, our method’s computational efficiency shines brightly, clocking an average processing time of just 5.22 s.

Elevating the foundational CGEM algorithm, our CGEMGA incarnation introduces a strategic optimization of the initial seed *s* through GA, coupled with the Mean Squared Residual (MSR) serving as a continual benchmark for each iteration. This innovation ensures not only a globally optimal output but also a refined selection of candidate gene modules, adeptly navigating the vast seas of RNA-seq data to pinpoint co-expressed genomes and their co-expression conditions with unprecedented efficiency and stability. The development of CGEMGA is shown to be superior to CGEM in terms of speed and its ability to link driver genes with breast cancer.

However, our voyage is not without its navigational buoys and potential horizons for expansion. The reliance on TCGA as the sole data harbor introduces a need for broader validation across diverse genomic databases. Moreover, the exclusive use of MSR as the guiding criterion beckons the exploration of additional metrics to enrich our biclustering search compass. Historical beacons, such as the scaling MSR (SMSR) introduced by Mukhopadhyay et al. in 2009 [46], and the biclustering with iterative sorting of weighted coefficients (BISWC) approach employed by Teng and Chan in 2008 [47], which meticulously prioritize and filter features based on their significance, hint at the vast potential of integrating multiple criteria to further refine our algorithm’s accuracy and relevance.

Furthermore, in their foundational work, Gunnar Carlsson and colleagues introduced the concept of topological data analysis (TDA), which offers insights into the underlying data structures and key learning processes, thereby facilitating improvements in deep-learning performance and generalization [48]. Tianyu Zhang and colleagues have proposed a multimodal deep-learning model that fuses mammography and ultrasound images with the objective of enhancing the precision of breast cancer molecular subtype prediction [49]. This approach focuses on the most pertinent features for the prediction task through an attention mechanism. Paul Gamble has developed a deep-learning system for the direct prediction of biomarker status in breast cancer tissues [50]. Moreover, he presents a methodology for enhancing the efficacy and precision of biomarker detection by elucidating the correlation between the morphological characteristics discerned by the model and the biomarker status through interpretable analysis. Xinmin Zhang provides a comprehensive overview of the current status and future direction of molecular classification in breast cancer, emphasizing the crucial role of molecular classification in individualized therapy [51]. Moreover, he presents the development of more accurate, reliable, and straightforward molecular classification methods. Lehmann examines two variants of the rs12976445 polymorphism of the *miR-125a* gene in breast cancer patients, investigating their correlation with breast cancer [52]. Furthermore, he elucidates the mechanism by which the U variant may diminish the expression level of *miR-125a* through online simulation. The findings of this research will provide new insights and ideas for the diagnosis and treatment of breast cancer. In their review, Yasmin Cura and colleagues considered the clinical relevance of genetic polymorphisms affecting the efficacy and safety of breast cancer treatments [53]. Moreover, they emphasized the importance of pharmacogenetic guidelines based on these polymorphisms and explored the development of more precise predictive models for individuals. The aforementioned methods illustrate that breast cancer prediction research is exploring innovative avenues that integrate bioinformatics concepts, including multimodal information fusion, deep-learning models, interpretability analysis, and molecular marker discovery, with bioinformatics techniques such as data mining, machine learning, network analysis, and computational biology. This represents the central focus of our future research endeavors.

In essence, the introduced CGEMGA algorithm serves not only as a model of efficiency and robustness in the pursuit of deciphering the genomic landscape of breast cancer but also as a validation of the transformative power of evolutionary algorithms in deciphering the intricate harmonies of gene expression patterns. In the future, our research will focus on incorporating a range of fitness functions as criteria for bicluster identification. This will allow us to refine the accuracy and enhance the pragmatic utility of our computational methodology. As we pursue this course of research, the practical implications of our findings encourage further investigation, suggesting a multitude of potential applications in the ongoing fight against breast cancer.

## Figures and Tables

**Table 1:** A comparative analysis of the characteristics of the six algorithms, namely CC, MCC, LAS, CGEM, RelDenClu, and CBSC.

Characteristics	Algorithms					
	CC	MCC	LAS	CGEM	RelDenClu	CBSC
Core Idea	Iterative spectral method for finding co-expressed gene and condition submatrices	Ensemble learning method combining multiple base biclustering algorithms for improved stability	Statistical method for finding large average submatrices in high-dimensional data, focusing on numerical features	Graph-based method for extracting conserved gene expression motifs	Find sets of observations with high local density	Find subspaces with high connectivity
Algorithm Type	Spectral clustering	Ensemble learning	Statistical method	Graph-based method	Density clustering	Connectivity clustering
Time Complexity	High	Moderate to high (depending on the number and type of base algorithms)	High	Moderate	High	High
Space Complexity	Moderate	High (requires storage of results from multiple base algorithms)	High (requires storage of extensive submatrix information)	Moderate	High	High
Applicable Data Type	Expression data	General (applicable to various types of data)	General (applicable to various types of data)	Expression data	Microarray data	High-dimensional data
Robustness	Moderate (sensitive to noise)	High (ensemble methods reduce the impact of noise)	High (statistical methods have some resistance to noise)	Moderate (depends on the stability of the graph structure)	High	High
Scalability	Moderate (suitable for medium to small-scale data)	Good (can be scaled to large-scale data)	Good (suitable for large-scale data)	Moderate (suitable for medium to small-scale data)	Moderate (suitable for medium to small-scale data)	Medium
Pattern Type Discovered	Co-expression patterns	Diverse patterns (depending on the base algorithms)	Numerically significant submatrices	Conserved expression motifs	Nonlinear relationships between features	Subspace structures based on connectivity
Real-world Applications	Gene expression analysis in bioinformatics	Widely applied in bioinformatics and machine learning	Bioinformatics, image processing, and other fields	Gene network analysis in bioinformatics	Gene functional grouping in microarray data	Gene functional grouping in bioinformatics

**Table 2:** Pseudo-code of CGEMGA algorithm.

**Algorithm 1** FINDMODULE(): algorithm for computing the largest module.
1. for *i* = 1 to *n_s_* do 2. **GA begin** 3. Create an initial population of *n* chromosomes *C_i_* (*i* = 1, 2, …, *n*) as seeds 4. Set iteration counter *t* = 0 5. Choose a subset *D_i_* of the samples with size *s_d_* 6. For every gene g in *D_i_*, include the pair (*g*, *s*) in the set *G_ij_* if g is in the state *s* in *c* and all *D_i_* samples 7. *C_ij_* = set of samples that agree with *c* in all the gene-states in *G_ij_* 8. Calculate the MSR fitness value for each chromosome 9. **while** (*t* < MAX) 10. Select a pair of chromosomes form initial population based on MSR fitness 11. Apply crossover operation on selected pair with crossover probability 12. Apply mutation on the offspring with mutation probability 13. Replace old population with newly generated population 14. Increment the current iteration *t* by 1. 15. **end while** 16. Discard (*C_ij_*, *G_ij_*) if *C_ij_* contains less than αn samples. 17. **returen** the best solution, *C_i_* with min MSR 18. **GA end** 19. **return** the module (C*, G*) that maximises |*G_ij_*|, 1 ≤ *i* ≤ *n_s_*

**Table 3:** Comparison of Fisher’s test *p-*values and MSR values of CGEM with our approach on 10 independent runs.

No.	Method			
	CGEM		CGEMGA	
	*p* Value	MSR Value	*p* Value	MSR Value
1	9.62 × 10^−6^	4.71 × 10^−2^	1.13 × 10^−4^	9.37 × 10^−2^
2	2.47 × 10^−5^	1.27 × 10^−1^	1.19 × 10^−4^	9.37 × 10^−2^
3	1.73 × 10^−4^	1.29 × 10^0^	1.25 × 10^−4^	9.37 × 10^−2^
4	1.84 × 10^−4^	1.44 × 10^0^	1.28 × 10^−4^	9.37 × 10^−2^
5	6.54 × 10^−4^	5.44 × 10^0^	1.55 × 10^−4^	9.37 × 10^−2^
6	1.20 × 10^−3^	6.89 × 10^0^	1.63 × 10^−4^	9.37 × 10^−2^
7	1.90 × 10^−3^	1.15 × 10	1.75 × 10^−4^	9.37 × 10^−2^
8	6.00 × 10^−3^	2.95 × 10	1.81 × 10^−4^	9.37 × 10^−2^
9	1.08 × 10^−2^	5.78 × 10	1.91 × 10^−4^	9.37 × 10^−2^
10	9.71 × 10^−2^	7.12 × 10^2^	1.91 × 10^−4^	9.37 × 10^−2^
Mean ± SD	1.18 × 10^−2^ ± 3.01 × 10^−2^	8.26 × 10 ± 2.21 × 10^2^	1.54 × 10^−4^ ± 3.06 × 10^−5^	9.37 × 10^−2^ ± 0

**Table 4:** Runtime of CGEM and our approach in seconds.

Method	Times (*n* = 10)										Mean ± SD
	1	2	3	4	5	6	7	8	9	10	
CGEM	1.89 × 10^3^	1.79 × 10^3^	1.77 × 10^3^	1.23 × 10^3^	1.21 × 10^3^	1.16 × 10^3^	1.13 × 10^3^	1.01 × 10^3^	9.59 × 10^2^	9.32 × 10^2^	1.31 × 10^3^ ± 3.66 × 10^2^
CGEMGA	4.94 × 10^0^	5.02 × 10^0^	5.11 × 10^0^	5.13 × 10^0^	5.25 × 10^0^	5.29 × 10^0^	5.30 × 10^0^	5.33 × 10^0^	5.39 × 10^0^	5.45 × 10^0^	5.22 × 10^0^ ± 1.65 × 10^−1^

**Table 5:** Fisher’s test *p-*values for 10 independent runs of the seven methods.

Method	Fisher’ Test *p* Values (*n* = 10)										Mean ± SD
	1	2	3	4	5	6	7	8	9	10	
MCC	1.50 × 10^−3^	7.30 × 10^−3^	9.20 × 10^−3^	1.03 × 10^−1^	1.75 × 10^−1^	4.31 × 10^−1^	5.09 × 10^−1^	6.67 × 10^−1^	8.98 × 10^−1^	9.03 × 10^−1^	3.70 × 10^−1^ ± 3.62 × 10^−1^
CC	1.50 × 10^−3^	7.30 × 10^−3^	4.99 × 10^−2^	1.74 × 10^−1^	1.78 × 10^−1^	1.95 × 10^−1^	4.12 × 10^−1^	5.59 × 10^−1^	6.73 × 10^−1^	8.54 × 10^−1^	3.10 × 10^−1^ ± 3.00 × 10^−1^
LAS	6.68 × 10^−5^	6.44 × 10^−4^	6.44 × 10^−4^	1.70 × 10^−3^	6.70 × 10^−3^	3.04 × 10^−2^	6.65 × 10^−2^	7.56 × 10^−2^	5.44 × 10^−1^	6.26 × 10^−1^	1.35 × 10^−1^ ± 2.40 × 10^−1^
CGEM	9.62 × 10^−6^	2.47 × 10^−5^	1.73 × 10^−4^	1.84 × 10^−4^	6.54 × 10^−4^	1.20 × 10^−3^	1.90 × 10^−3^	6.00 × 10^−3^	1.08 × 10^−2^	9.71 × 10^−2^	1.18 × 10^−2^ ± 3.01 × 10^−2^
RelDenClu	1.40 × 10^−68^	1.43 × 10^−67^	1.34 × 10^−34^	1.35 × 10^−34^	6.33 × 10^−34^	3.05 × 10^−13^	7.49 × 10^−13^	1.44 × 10^−8^	1.45 × 10^−2^	2.01 × 10^−2^	3.46 × 10^−3^ ± 6.98 × 10^−3^
CBSC	1.54 × 10^−13^	1.57 × 10^−13^	1.48 × 10^−12^	3.35 × 10^−11^	1.48 × 10^−10^	6.97 × 10^−10^	8.23 × 10^−6^	1.58 × 10^−4^	1.60 × 10^−4^	2.21 × 10^−2^	2.24 × 10^−3^ ± 7.41 × 10^−3^
CGEMGA	1.13 × 10^−4^	1.19 × 10^−4^	1.25 × 10^−4^	1.28 × 10^−4^	1.55 × 10^−4^	1.63 × 10^−4^	1.75 × 10^−4^	1.81 × 10^−4^	1.91 × 10^−4^	1.91 × 10^−4^	1.54 × 10^−4^ ± 3.06 × 10^−5^

**Table 6:** The gene find by our approach form the TCGA breast cancer data.

No.	Gene Symbol	Name	Cytogenetic Band	No.	Gene Symbol	Name	Cytogenetic Band
1	*MTOR*	mechanistic target of rapamycin	1p36.22	24	*SDHAF2*	succinate dehydrogenase complex assembly factor 2	11q12.2
2	*SF3B1*	splicing factor 3b, subunit 1, 155 kDa	2q33.1	25	*KDM5A*	lysine (K)-specific demethylase 5A, JARID1A	12p13.33
3	*POLQ*	DNA polymerase theta	3q13.33	26	*PRPF40B*	pre-mRNA processing factor 40 homolog B	12q13.12
4	*MECOM*	MDS1 and EVI1 complex locus	3q26.2	27	*NCOR2*	nuclear receptor corepressor 2	12q24.31
5	*TET2*	tet oncogene family member 2	4q24	28	*RAD51B*	RAD51 paralog B	14q24.1
6	*FAT1*	FAT atypical cadherin 1	4q35.2	29	*TCL1A*	T-cell leukemia/lymphoma 1A	14q32.13
7	*TLX3*	T-cell leukemia, homeobox 3 (HOX11L2)	5q35.1	30	*DROSHA*	drosha ribonuclease III	15p13.3
8	*SRSF3*	serine/arginine-rich splicing factor 3	6p21.31	31	*CHD2*	chromodomain helicase DNA binding protein 2	15q26.1
9	*DEK*	DEK oncogene (DNA binding)	6p22.3	32	*PRKCB*	protein kinase C beta	16p12.2
10	*SGK1*	serum/glucocorticoid regulated kinase 1	6q23.2	33	*RMI2*	RecQ mediated genome instability 2	16p13.13
11	*EZR*	ezrin	6q25.3	34	*CDH1*	cadherin 1, type 1, E-cadherin (epithelial) (ECAD)	16q22.1
12	*MACC1*	MET transcriptional regulator MACC1	7p21.1	35	*TP53*	tumor protein p53	17p13.1
13	*SBDS*	Shwachman-Bodian-Diamond syndrome protein	7q11.21	36	*KAT7*	lysine acetyltransferase 7	17q21.33
14	*CUX1*	cut-like homeobox 1	7q22.1	37	*SRSF2*	serine/arginine-rich splicing factor 2	17q25.2
15	*KAT6A*	K(lysine) acetyltransferase 6A	8p11.21	38	*KDSR*	3-ketodihydrosphingosine reductase	18q21.33
16	*GNAQ*	guanine nucleotide binding protein (Gprotein), q polypeptide	9q21.2	39	*CEP89*	centrosomal protein 89 kDa	19q13.11
17	*CNTRL*	centriolin	9q33.2	40	*ARHGAP35*	Rho GTPase activating protein 35	19q13.32
18	*LARP4B*	La ribonucleoprotein domain family member 4B	10p15.3	41	*TOP1*	topoisomerase (DNA) I	20q12
19	*A1CF*	APOBEC1 complementation factor	10q11.23	42	*KDM5C*	lysine (K)-specific demethylase 5C (JARID1C)	Xp11.22
20	*KAT6B*	K(lysine) acetyltransferase 6B	10q22.2	43	*KDM6A*	lysine (K)-specific demethylase 6A, UTX	Xp11.3
21	*NUP98*	nucleoporin 98kDa	11p15.4	44	*TMSB4X*	Thymosin Beta 4 X-Linked	Xp22.2
22	*CLP1*	cleavage and polyadenylation factor I subunit 1	11q12.1	45	*CRLF2*	cytokine receptor-like factor 2	Xp22.33
23	*FEN1*	flap structure-specific endonuclease 1	11q12.2				

**Table 7:** The functional enrichment analysis results of the discovered drivers of our approach.

Term	Percentage	*p*-Value	FDR
hsa05205:Proteoglycans in cancer	11.1	3.8 × 10^−3^	5.98 × 10^−1^
hsa05214:Glioma	6.7	2.4 × 10^−2^	7.75 × 10^−1^
hsa04971:Gastric acid secretion	6.7	2.4 × 10^−2^	7.75 × 10^−1^
hsa05200:Pathways in cancer	13.3	2.4 × 10^−2^	7.75 × 10^−1^
h_pkcPathway:Activation of PKC through G protein coupled receptor	4.4	3.0 × 10^−2^	7.53 × 10^−1^
hsa03040:Spliceosome	8.9	3.1 × 10^−2^	7.75 × 10^−1^
hsa05163:Human cytomegalovirus infection	8.9	3.4 × 10^−2^	7.75 × 10^−1^
hsa04670:Leukocyte transendothelial migration	6.7	5.1 × 10^−2^	7.75 × 10^−1^
hsa04935:Growth hormone synthesis, secretion and action	6.7	5.6 × 10^−2^	7.75 × 10^−1^
hsa04071:Sphingolipid signaling pathway	6.7	5.6 × 10^−2^	7.75 × 10^−1^
hsa04919:Thyroid hormone signaling pathway	6.7	5.6 × 10^−2^	7.75 × 10^−1^
h_myosinPathway:PKC-catalyzed phosphorylation of inhibitory phosphoprotein of myosin phosphatase	4.4	5.9 × 10^−2^	7.53 × 10^−1^
h_ccr5Pathway:Pertussis toxin-insensitive CCR5 Signaling in Macrophage	4.4	7.1 × 10^−2^	7.53 × 10^−1^
hsa04371:Apelin signaling pathway	6.7	7.2 × 10^−2^	7.75 × 10^−1^
hsa05206:MicroRNAs in cancer	8.9	7.4 × 10^−2^	7.75 × 10^−1^
hsa05017:Spinocerebellar ataxia	6.7	7.6 × 10^−2^	7.75 × 10^−1^
h_calcineurinPathway:Effects of calcineurin in Keratinocyte Differentiation	4.4	7.9 × 10^−2^	7.53 × 10^−1^
hsa05226:Gastric cancer	6.7	8.1 × 10^−2^	7.75 × 10^−1^
hsa04150:mTOR signaling pathway	6.7	8.8 × 10^−2^	7.75 × 10^−1^
h_par1pathway:Thrombin signaling and protease-activated receptors	4.4	9.1 × 10^−2^	7.53 × 10^−1^
h_chemicalPathway:Apoptotic Signaling in Response to DNA Damage	4.4	9.1 × 10^−2^	7.53 × 10^−1^
h_ccr3Pathway:CCR3 signaling in Eosinophils	4.4	9.5 × 10^−2^	7.53 × 10^−1^
h_eif4Pathway:Regulation of eIF4e and p70 S6 Kinase	4.4	9.9 × 10^−2^	7.53 × 10^−1^
h_cxcr4Pathway:CXCR4 Signaling Pathway	4.4	9.9 × 10^−2^	7.53 × 10^−1^
hsa05225:Hepatocellular carcinoma	6.7	1.0 × 10^−1^	7.75 × 10^−1^

## Data Availability

The datasets analyzed for this study can be found in the TCGA and cBioPortal, https://www.cancer.gov/ccg/research/genome-sequencing/tcga (accessed on 15 November 2022) and http://www.cbioportal.org/ (accessed on 15 November 2022).

## Supplementary Materials

The following supporting information can be downloaded at: https://www.mdpi.com/article/10.3390/biomedicines12092086/s1. Figure S1: Runtime of CGEM and CGEMGA in seconds.
