# Supplementary material for: Evolutionary Mechanism Based Conserved Gene Expression Biclustering Module Analysis for Breast Cancer Genomics
Source: Biomedicines. 2024 Sep 12;12(9):2086. doi: 10.3390/biomedicines12092086 (PMC11428256; doi:10.3390/biomedicines12092086)
Supplement: Supplementary file 1 [file biomedicines-12-02086-s001.zip › biomedicines-3095827-supplementary.pdf]

Additional File S1 –Supplementary Figures

Evolutionary Mechanism Based Conserved Gene Expression Biclustering Module Analysis for Breast Cancer Genomics

Wei Yuan, Yaming Li, Zhengpan Han, Yu Chen, Jinnan Xie, Jianguo Chen, Zhisheng Bi * and Jianing Xi *

School of Biomedical Engineering, Guangzhou Medical University, Guangzhou 511436, China
* Correspondence: bivictor@gmail.com (Z.B.); xjn@gzhmu.edu.cn (J.X.)

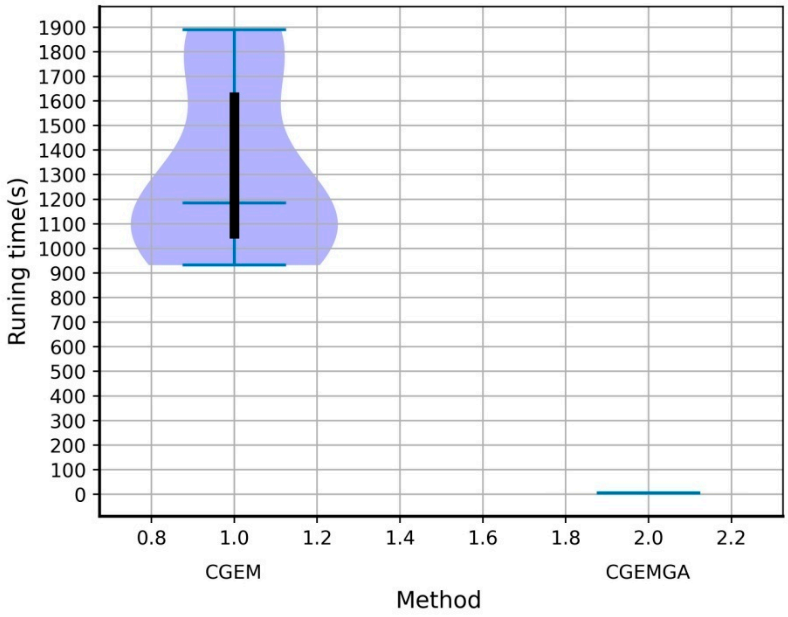

Figure S1. Runtime of CGEM and CGEMGA in seconds.
